# Body mass index and all cause mortality in HUNT and UK Biobank studies: linear and non-linear mendelian randomisation analyses

**DOI:** 10.1136/bmj.l1042

**Published:** 2019-03-26

**Authors:** Yi-Qian Sun, Stephen Burgess, James R Staley, Angela M Wood, Steven Bell, Stephen K Kaptoge, Qi Guo, Thomas R Bolton, Amy M Mason, Adam S Butterworth, Emanuele Di Angelantonio, Gunnhild Å Vie, Johan H Bjørngaard, Jonas Minet Kinge, Yue Chen, Xiao-Mei Mai

**Affiliations:** 1 Department of Clinical and Molecular Medicine (IKOM), NTNU, Norwegian University of Science and Technology, Trondheim, Norway; 2 MRC Biostatistics Unit, Cambridge Institute of Public Health, Cambridge CB2 0SR, UK; 3 Cardiovascular Epidemiology Unit, Department of Public Health and Primary Care, University of Cambridge, Cambridge, UK; 4 MRC Integrative Epidemiology Unit, Population Health Sciences, Bristol Medical School, University of Bristol, Bristol, UK; 5 NIHR Blood and Transplant Research Unit in Donor Health and Genomics, Department of Public Health and Primary Care, University of Cambridge, Cambridge, UK; 6 Department of Public Health and Nursing, NTNU, Norwegian University of Science and Technology, Trondheim, Norway; 7 Norwegian Institute of Public Health, Oslo, Norway; 8 University of Oslo, Oslo, Norway; 9 School of Epidemiology and Public Health, Faculty of Medicine, University of Ottawa, Ottawa, ON, Canada

## Abstract

Editor’s Note on Methodological Criticism and an Updated Analysis

**Objective:**

To investigate the shape of the causal relation between body mass index (BMI) and mortality.

**Design:**

Linear and non-linear mendelian randomisation analyses.

**Setting:**

Nord-Trøndelag Health (HUNT) Study (Norway) and UK Biobank (United Kingdom).

**Participants:**

Middle to early late aged participants of European descent: 56 150 from the HUNT Study and 366 385 from UK Biobank.

**Main outcome measures:**

All cause and cause specific (cardiovascular, cancer, and non-cardiovascular non-cancer) mortality.

**Results:**

12 015 and 10 344 participants died during a median of 18.5 and 7.0 years of follow-up in the HUNT Study and UK Biobank, respectively. Linear mendelian randomisation analyses indicated an overall positive association between genetically predicted BMI and the risk of all cause mortality. An increase of 1 unit in genetically predicted BMI led to a 5% (95% confidence interval 1% to 8%) higher risk of mortality in overweight participants (BMI 25.0-29.9) and a 9% (4% to 14%) higher risk of mortality in obese participants (BMI ≥30.0) but a 34% (16% to 48%) lower risk in underweight (BMI <18.5) and a 14% (−1% to 27%) lower risk in low normal weight participants (BMI 18.5-19.9). Non-linear mendelian randomisation indicated a J shaped relation between genetically predicted BMI and the risk of all cause mortality, with the lowest risk at a BMI of around 22-25 for the overall sample. Subgroup analyses by smoking status, however, suggested an always-increasing relation of BMI with mortality in never smokers and a J shaped relation in ever smokers.

**Conclusions:**

The previously observed J shaped relation between BMI and risk of all cause mortality appears to have a causal basis, but subgroup analyses by smoking status revealed that the BMI-mortality relation is likely comprised of at least two distinct curves, rather than one J shaped relation. An increased risk of mortality for being underweight was only evident in ever smokers.

## Introduction

Body mass index (BMI) is a commonly used simple measure that combines weight and height to classify obesity.^1^ Over the past decades, the prevalence of obesity (defined as a BMI of ≥30.0) has increased worldwide.^2^ Although many studies have suggested that obesity increases the risks of several adverse health conditions,^3^ life expectancy during the same period has increased.^4^ Several meta-analyses have shown a J shaped relation between BMI and all cause mortality, with the lowest point of the curve in the normal weight (BMI 18.5-24.9) or even the overweight (25.0-29.9) category.^5^- ^9^ However, observational results, even from well designed studies with large numbers of participants, can be biased by residual confounding and reverse causation. This could explain the increased risk of mortality observed in underweight (BMI <18.5) people. Therefore, investigating the shape of the causal relation between BMI and all cause mortality is of great interest.

One approach for investigating this is mendelian randomisation, in which the association between a disease outcome and genetically predicted values of a modifiable risk factor are considered.^10^ The rationale for considering genetically predicted values is that the genetic code is fixed at conception and is therefore somewhat immune to the influence of both confounding and reverse causation. Under the assumptions that participants with genetic variants predisposing them to higher levels of the risk factor are similar on average to participants with genetic variants predisposing them to lower levels of the risk factor, and that genetic variants only influence the outcome through their association with the risk factor (here BMI), mendelian randomisation provides unconfounded estimates representing average changes in the outcome for lifelong differences in BMI values.^11^ If values of the risk factor can be altered in a way that reflects these genetic differences, then these estimates have a causal interpretation.^12^ See Davies and colleagues for a recent review of the approach.^13^

We applied mendelian randomisation to investigate the potential causal relation of BMI on all cause mortality in two population based prospective cohorts: the Norwegian Nord-Trøndelag Health (HUNT) Study and UK Biobank. Linear analyses were carried out to quantify the average causal effect of a population shift in the BMI distribution, and non-linear analyses to characterise the shape of the BMI-mortality relation. Subgroup analyses were performed stratifying by sex, smoking status, and age at risk. We also investigated the shape of the relation of BMI with disease specific mortality and morbidity.

## Methods

### The HUNT Study

We used data from the second wave (1995-97) of the HUNT Study on 65 229 people living in Nord-Trøndelag aged 20 and older.^14^ Participants were followed up until 15 April 2015 or their date of death. We excluded participants without data on BMI or genetic variants for BMI, leaving 56 150 people for analysis. Data on baseline variables were collected by self administered questionnaires or clinical examination. Trained nurses measured height and weight at the clinical examination, with the participants wearing light clothes and no shoes. Height was measured in whole centimetres. Weight was measured to the nearest 0.5 kg. Genome-wide genotyping was carried out by using Illumina HumanCoreExome arrays.

### UK Biobank

The UK Biobank cohort comprises around 500 000 people (94% of self reported European ancestry) aged 40 to 69 at baseline and recruited between 2006 and 2010 in 22 assessment centres throughout the UK. Participants were followed up until 17 February 2016 or their date of death.^15^ The database contains genome-wide genotyping of baseline samples from all participants, results of clinical examinations, assays of biological samples, and detailed information on self reported health behaviour, and is supplemented by linkage with electronic health records such as hospital inpatient data, mortality data, and cancer registries. Data on height and weight were collected at baseline when participants attended the assessment centre. Height was measured in whole centimetres with a Seca 202 device. Weight was measured to the nearest 0.1 kg.

We performed detailed quality control procedures on UK Biobank participants and on genetic variants. In total, 366 385 unrelated participants of European ancestry were included in the analyses. Further details on both studies are provided in the supplementary material.

### Single nucleotide polymorphisms and allele score as instrumental variables

We selected 77 single nucleotide polymorphisms as candidate instrumental variables for BMI based on European sex-combined analyses in a genome-wide association study of the GIANT (Genetic Investigation of Anthropometric Traits) consortium.^16^ Two of these variants (rs12016871 and rs2033732) were not available in the HUNT Study, and a further two variants (rs13021737 and rs16951275) were excluded from the analyses owing to an association with smoking status in the HUNT Study. We calculated an externally weighted allele score for each participant by multiplying the number of BMI-increasing alleles the participant carried by the variant’s association with BMI from the GIANT study (see supplementary table 1) and summing across the remaining 73 variants. Overall, the weighted allele score explained 2.0% and 1.6% of the variance in BMI in the HUNT Study and the UK Biobank, respectively, corresponding to F statistics of 1121 and 5964.

### Study design

We performed several mendelian randomisation analyses, assessing the association between genetically predicted BMI and mortality outcomes or disease incidence. When the relation between the exposure and the outcome is non-linear, a linear mendelian randomisation estimate represents the average change in the outcome resulting from a shift in the population distribution of the exposure.^17^ Here, we express estimates for each 1 unit increase in BMI. We also performed non-linear mendelian randomisation analyses to estimate the shape of the association between genetically predicted BMI and the outcome.^18^

Our primary analysis considered all cause mortality as the outcome. We also conducted a priori specified subgroup analyses considering men and women, never smokers and ever smokers, and younger and older participants (age at risk <65 years and ≥65 years). In addition, we studied associations with cause specific mortality events (cardiovascular, cancer, and other) and with incident diseases (cardiovascular and cancer) in the UK Biobank.

### Statistical analyses

We calculated linear mendelian randomisation estimates for BMI on the risk of mortality by using the ratio of coefficients method.^19^ Linear regression was used to estimate the association of the allele score with BMI and Cox proportional hazards regression to estimate the association of the allele score with mortality. We adjusted for age, sex, centre (in UK Biobank), and for age-squared (in linear regression). Estimates were also calculated within categories of residual BMI (<18.5, 18.5-19.9, 20.0-24.9, 25.0-29.9, and ≥30.0). This categorisation is based on World Health Organization guidelines.^1^ By stratifying on residual BMI, defined as a participant’s BMI minus the centred genetic contribution to BMI from variants included in the allele score, we compared individuals in the population who would have similar BMI values (that is, values in the same stratum) if they had the same genetic code. Stratifying on BMI directly would distort estimates, as BMI is on the causal pathway from the genetic variants to the outcome. As sensitivity analyses, we performed the MR-Egger method,^20^ with an intercept term differing from zero representing evidence of directional pleiotropy, and the weighted median method,^21^ which is less sensitive to genetic variants having outlying variant-specific causal estimates. We also generated a scatterplot as a visual check for outliers in the variant-specific causal estimates, as such variants might be pleiotropic.

**Fig 1:**
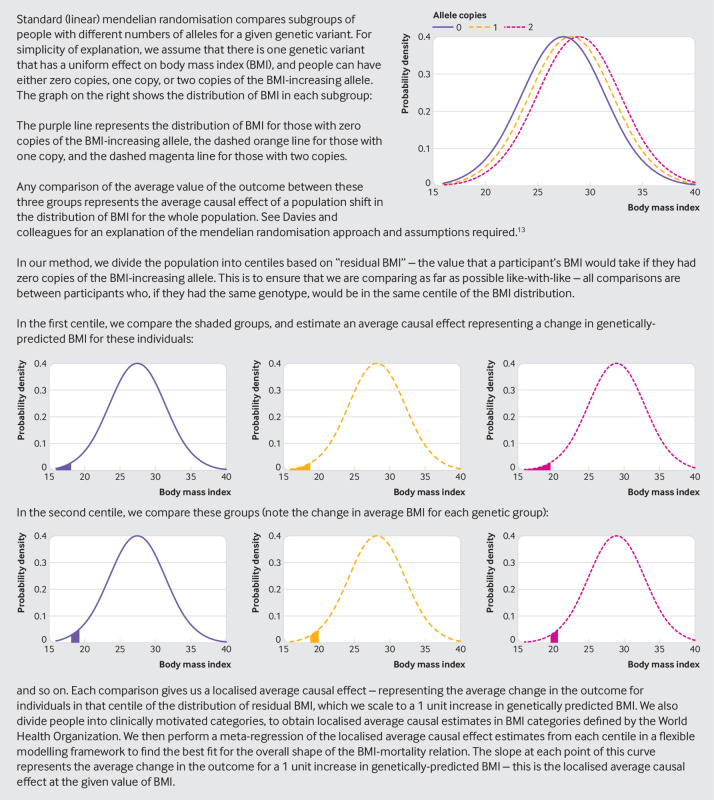
Description of method for estimating shape of body mass index-mortality relation by using genetic variants

We applied a fractional polynomial method to calculate non-linear mendelian randomisation estimates of BMI on the risk of all cause mortality.^17^ ^18^ Briefly, we divided the sample into 100 stratums by using residual BMI. Then we calculated the linear mendelian randomisation estimate, referred to as a localised average causal effect, in each stratum of the population as a ratio of coefficients: the association of the allele score with the outcome divided by the association of the allele score with the exposure. We performed meta-regression of the localised average causal effect estimates against the mean of the exposure in each stratum in a flexible semiparametric framework by using the derivative of fractional polynomial models of degrees 1 and 2. Two tests for non-linearity are reported: a trend test, which assesses for a linear trend among the localised average causal effect estimates, and a fractional polynomial test, which assesses whether a non-linear model fits the localised average causal effect estimates better than a linear model. Figure 1 provides an intuitive explanation of the method. 

All non-linear comparisons are conducted within stratums of the population defined by residual BMI, so they only provide meaningful information on comparisons within these stratums. Hence we encourage focusing on the slope of the BMI-mortality relation at different values of the BMI distribution, rather than differences that extrapolate across the whole range of the distribution. The slope of the graph of the BMI-mortality relation is the average causal estimate at that value of BMI. A statistically significant causal estimate at a particular BMI value is evidenced not when the confidence interval for the hazard ratio excludes the value 1, but when the slopes of the upper and lower bounds of its confidence interval are both positive for a positive estimate, or both negative for a negative estimate.

All statistical analyses were performed with R (version 3.4.3) or Stata/SE 15.1 (StataCorp, College Station, TX). The supplementary material provides a detailed description of the methods.

### Patient and public involvement

No patients were involved in setting the research question or the outcome measures, nor were they involved in the design or implementation of the study. No patients were asked to advise on interpretation or writing up of results. There are no plans to disseminate the results of the research to study participants or the relevant patient community.

## Results

Table 1 shows the baseline characteristics of the participants. Participants in the HUNT Study were younger at baseline (mean age 49.6 *v* 56.7) and had a slightly lower mean BMI (26.3 *v* 27.4) than those in the UK Biobank. Distributions of BMI in the HUNT Study and UK Biobank were similar and approximately normal (supplementary table 2), except there were more extremely obese participants in the UK Biobank. The HUNT Study had a longer follow-up (median 18.5 *v* 7.0 years) and more deaths (12 015 *v* 10 344). A greater proportion of participants in the HUNT Study were ever smokers than in the UK Biobank (55.9% *v* 46.1%).

**Table 1:** Baseline characteristics of participants in the HUNT Study and UK Biobank

Characteristics	HUNT Study	UK Biobank
No of participants	56 150	366 385
No (%) of men	26 447 (47.1)	168 171 (45.9)
Mean (SD) age at baseline (years)	49.6 (16.6)	56.7 (8.0)
No of deaths	12 015	10 344
Mean (SD) body mass index	26.3 (4.1)	27.4 (4.8)
Median follow-up (years)	18.5	7.0
No (%) of ever smokers	31 388 (55.9)	168 903 (46.1)

### Linear mendelian randomisation analyses

Table 2 shows that linear mendelian randomisation analyses provided some evidence of an overall association between genetically predicted BMI and all cause mortality, suggesting that increasing the overall distribution of BMI in the population by 1 unit would lead to an overall increase in the risk of mortality of 4% (95% confidence interval 2% to 6%). The estimate was larger in women than in men for both studies. However, opposite directions of association were seen between BMI categories, with a 1 unit increase in genetically predicted BMI leading to a 5% (95% confidence interval 1% to 8%) higher risk of mortality in overweight participants (BMI 25.0-29.9) and a 9% (4% to 14%) higher risk of mortality in obese participants (BMI ≥30.0), but a 34% (16% to 48%) lower risk in underweight participants (BMI <18.5) and a 14% (−1% to 27%) lower risk in low normal weight participants (BMI 18.5-19.9) (P value for trend 0.05 in HUNT Study, 0.02 in UK Biobank). The MR-Egger test did not detect substantial directional pleiotropy (MR-Egger intercept 0.005, P=0.13 in HUNT Study; −0.002, P=0.71 in UK Biobank). The MR-Egger and weighted median methods gave similar results to the primary linear analysis method in UK Biobank (supplementary table 3), and the scatterplot did not identify any outlying genetic variants (supplementary fig 1). In the HUNT Study, estimates from the robust methods were substantially attenuated towards the null. One outlier was detected, although omitting this variant from the analyses did not materially affect our findings.

**Table 2:** Linear mendelian randomisation estimates. Hazard ratios for all cause mortality for each 1 unit increase in body mass index (BMI)

All cause mortality	HUNT Study			UK Biobank			Overall	
	Hazard ratio (95% CI)	P value		Hazard ratio (95% CI)	P value		Hazard ratio (95% CI)	P value
Overall	1.03 (1.00 to 1.06)	0.09		1.05 (1.02 to 1.09)	0.002		1.04 (1.02 to 1.06)	<0.001
Men	1.01 (0.96 to 1.06)	0.75		1.05 (1.01 to 1.10)	0.03		1.04 (1.00 to 1.07)	0.05
Women	1.05 (1.00 to 1.09)	0.03		1.06 (1.01 to 1.11)	0.03		1.05 (1.02 to 1.09)	0.002
Within residual BMI categories:								
Underweight (<18.5)	0.79 (0.56 to 1.13)	0.19		0.57 (0.41 to 0.79)	<0.001		0.66 (0.52 to 0.84)	<0.001
Low normal weight (18.5-19.9)	0.90 (0.71 to 1.14)	0.40		0.82 (0.66 to 1.03)	0.09		0.86 (0.73 to 1.01)	0.07
High normal weight (20.0-24.9)	0.98 (0.93 to 1.04)	0.61		1.00 (0.94 to 1.06)	0.98		0.99 (0.95 to 1.04)	0.07
Overweight (25.0-29.9)	1.04 (0.99 to 1.09)	0.10		1.05 (1.00 to 1.11)	0.04		1.05 (1.01 to 1.08)	0.01
Obese (≥30.0)	1.05 (0.98 to 1.13)	0.14		1.11 (1.05 to 1.18)	<0.001		1.09 (1.04 to 1.14)	<0.001
Trend test	NA	0.05		NA	0.02		NA	0.01

NA=not applicable.

### Non-linear mendelian randomisation analyses

**Fig 2:**
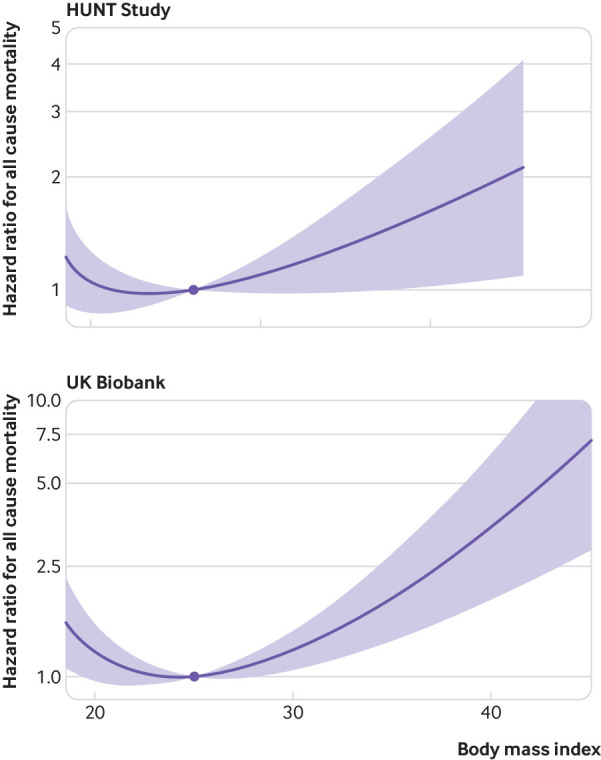
Non-linear mendelian randomisation. Dose-response curve between body mass index and all cause mortality for HUNT Study and UK Biobank. Gradient at each point of the curve is the localised average causal effect. Shaded areas represent 95% confidence intervals

Figure 2 shows that the overall results were similar between the HUNT Study and UK Biobank. We observed a J shaped relation between genetically predicted BMI and all cause mortality. The curved shape of the relation was more pronounced in UK Biobank—with higher risk both in underweight participants and in overweight or obese participants. The lowest risk for the overall population was at a BMI of around 22-23 in the HUNT Study and around 25 in UK Biobank. Similar results were observed in sensitivity analyses omitting participants with a mortality event in the first two years of follow-up (supplementary fig 2).

### Subgroup analyses

**Fig 3:**
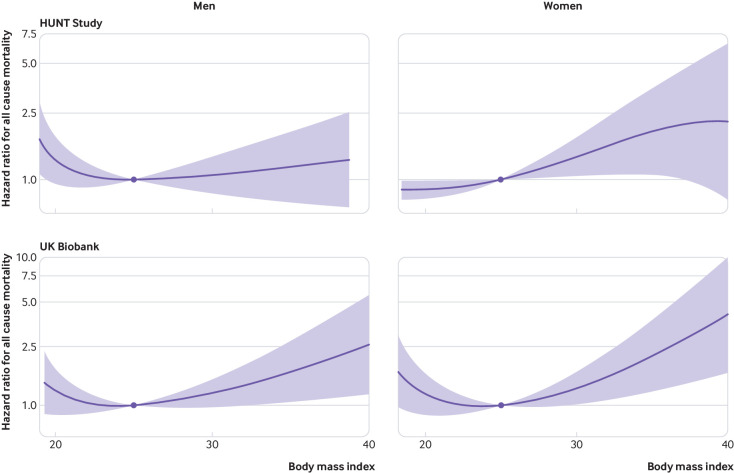
Non-linear mendelian randomisation. Dose-response curve between body mass index and all cause mortality in men and women for HUNT Study and UK Biobank. Gradient at each point of the curve is the localised average causal effect. Shaded areas represent 95% confidence intervals

Figure 3 shows the analysis split by sex. The slope for greater harm of increasing BMI among overweight or obese participants was more evident in women than in men.

**Fig 4:**
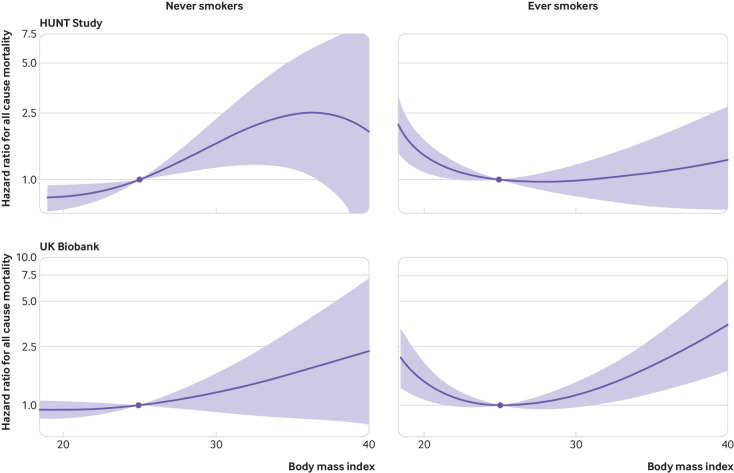
Non-linear mendelian randomisation. Dose-response curve between body mass index and all cause mortality in never smokers and ever smokers for HUNT Study and UK Biobank. Gradient at each point of the curve is the localised average causal effect. Shaded areas represent 95% confidence intervals

Figure 4 shows the analyses stratified by smoking status. The shape of the BMI-mortality relation was markedly different between never smokers and ever smokers. In never smokers, the shape of the dose-response relation was always-increasing in both studies, with no evidence for a harmful effect of reducing BMI in underweight participants (supplementary table 4). The increase in risk of all cause mortality with increasing BMI for never smokers was most clear in the HUNT Study, with a positive slope throughout the underweight, normal weight, and overweight categories. In UK Biobank, the shape of the relation was similar, although confidence intervals were wide and compatible with a null effect at all values of BMI. In ever smokers, the relation was J shaped in both studies, with a clear detrimental effect of reduced BMI in the underweight category and the low normal weight category. In severely obese participants in the HUNT Study, there was no increased risk of all cause mortality associated with higher BMI, possibly owing to fewer people having a BMI greater than 35.0. In the analyses split by age at risk (supplementary fig 3), the harmful effect of low BMI on mortality was clearer in younger participants. However, ever smokers comprised a considerable proportion of the deaths before age 65 (75% in HUNT Study and 60% in UK Biobank), meaning that differences in the shape of the dose-response relation between the age categories could be explained by smoking status.

**Fig 5:**
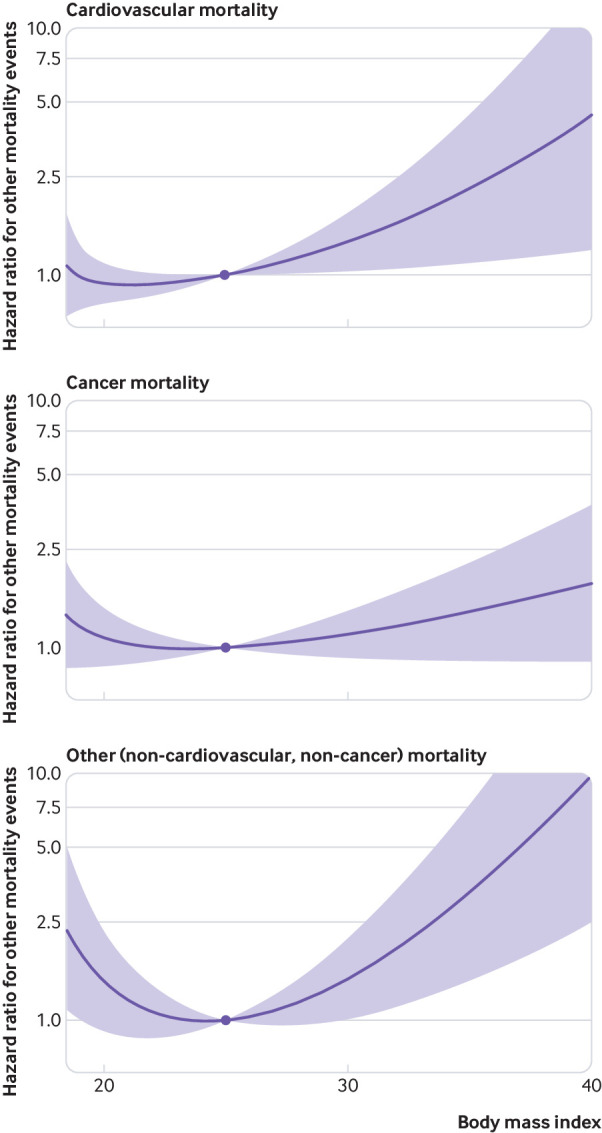
Non-linear mendelian randomisation in UK Biobank. Dose-response curve between body mass index and cause specific mortality. Gradient at each point of the curve is the localised average causal effect. Shaded areas represent 95% confidence intervals

Figure 5 shows the analyses for cause specific mortality in UK Biobank. The BMI-mortality curve for cardiovascular mortality (2145 deaths) was increasing, with increased risk associated with a higher BMI in the overweight and obese categories, no clear evidence for harm of lower BMI in the underweight category, and the lowest risk of mortality at a BMI of around 21-22. In contrast, the BMI-mortality curve for cancer mortality (6125 deaths) was flatter, with no strong evidence that BMI affects cancer mortality in any BMI category. Finally, the dose-response relation for other causes of mortality (non-cardiovascular non-cancer, 1998 deaths) had a profoundly curved J shape, with the lowest risk of mortality at a BMI of 23.0-24.0. The main causes of death in the “other” group were respiratory diseases (27%); diseases of the digestive system, including alcoholic liver disease (18%); diseases of the nervous system (15%); and deaths from external causes, including suicide (11%). In analyses for incident diseases in UK Biobank (supplementary fig 4), the curve for the relation between BMI and cardiovascular disease (7087 events) was increasing but with a much shallower slope that was compatible with a null effect, while the curve for any cancer (24 667 events) was again flat.

Supplementary tables 5 and 6 show information on mortality events in subgroups. In the UK Biobank study, trend and fractional polynomial tests suggested non-linear relations overall and in most subgroup analyses, but not for never smokers (supplementary table 7). Supplementary tables 8 and 9 provide estimates of the hazard ratio for centiles of the BMI distribution.

## Discussion

In this mendelian randomisation study of two large prospective population based cohorts, we found an overall J shaped relation between genetically predicted BMI and the risk of all cause mortality. The lowest risk was at a BMI of around 22-25. Risk of mortality was increased both in underweight participants and in overweight and obese participants. These results are similar to those from the most recent and largest observational meta-analyses.^5^ ^6^ ^7^ However, subgroup analyses revealed that the overall shape of the BMI-mortality relation comprised distinct curves rather than being one J shaped relation.

In the analyses stratified by smoking status, the BMI-mortality relation was always-increasing in never smokers with no evidence of harm of lower BMI in underweight participants. In contrast, a J shaped (or even U shaped) BMI-mortality relation was observed in ever smokers, with estimates suggesting a harmful effect of lower BMI in the underweight and normal weight categories. Similarly, the BMI-mortality relation was J shaped or decreasing in younger participants (<65 years), but it was generally increasing in older participants (>65 years). This is not consistent with results of several observational studies in older people, in which overweight categories were associated with a lower risk of all cause mortality.^22^ ^23^ There is an intrinsic limitation in separating age and smoking status, as deaths before age 65 were more common in ever smokers.

Another factor that is difficult to separate is cause specific mortality, as both never smokers and ever smokers and younger and older participants, differed substantially in their distributions of cause of death. We found an increasing relation between BMI and cardiovascular mortality, a null relation for cancer mortality, and a steep U shaped relation for non-cardiovascular non-cancer mortality.

### Possible explanations for findings

The mechanisms leading to increased all cause mortality might be different in underweight and overweight or obese participants. Underweight status—or its underlying causes, such as malnutrition—could lead to decreased immune function and an increased risk of infection.^24^ Underweight people might have an increased risk of surgical complications.^25^ Moreover, being underweight is associated with psychological disorders. A previous systematic review showed that being underweight was associated with an increased risk of completed suicide.^26^ A recent observational study reported a J shaped association between BMI and all cause mortality and a more profound U shaped association between lean body mass and mortality,^27^ suggesting that the higher risk of all cause mortality in the lower range of BMI might be explained by low lean mass rather than low fat mass. Low fat-free mass has also been reported to associate more strongly with the risk of all cause mortality than low fat mass.^28^

For a given BMI, women have higher levels of subcutaneous adipose tissue and fat mass than men,^29^ which could explain the steeper curve for harm of BMI increases in overweight and obese women compared with men in both the HUNT Study and UK Biobank.

The relation between smoking and obesity is complex, with previous evidence showing statistical interaction in their relation with mortality consistent with competitive antagonism.^30^ People with higher genetically predicted BMI are more likely to be smokers.^31^ However, smoking also reduces body weight.^32^ Increased mortality in underweight smokers might be driven by respiratory diseases,^33^ a major component of non-cardiovascular non-cancer mortality. This could explain the differences in the BMI-mortality relation between ever smokers and never smokers, as respiratory diseases are more common in ever smokers.

### Strengths and limitations of this study

Our study explored the shape of the potential causal relation between BMI and the risk of all cause mortality in a mendelian randomisation framework using fractional polynomial methodology. This method enables the division of the sample population into fine stratums, as stratum specific estimates are smoothed to give an overall BMI-mortality curve. A fine stratification is crucial to investigate the effect of lower BMI in underweight participants (1% to 3% of our sample populations).

Our study is limited by the study type. Compared with observational studies, mendelian randomisation studies are less vulnerable to bias from reverse causation and unmeasured confounding, particularly relating to confounding factors acting after the genetic code is fixed at conception. The genetic variants were not associated with important confounders such as smoking and socioeconomic status in the HUNT Study. The genetic variants might be subject to residual confounding or pleiotropy. However, the MR-Egger test did not detect substantial directional pleiotropy, neither did the MR-Egger or weighted median method produce a substantially different result in UK Biobank. Our investigation is observational rather than interventional, so a more conservative interpretation that the results represent unconfounded estimates, rather than causal estimates, could be preferred. However, if interventions in BMI can be conceived that are equivalent to how the genetic variants influence BMI, then a causal interpretation is warranted. Our results could be affected by collider bias. Stratifying on residual BMI rather than BMI directly avoids bias in the overall analysis, but stratifying on smoking status leads to collider bias. Given the size of the effect of BMI on smoking status previously observed in UK Biobank,^31^ however, the magnitude of collider bias in this case is likely to be negligible.^34^

A further limitation is the possibility of ascertainment bias. About 30% of the inhabitants in Nord-Trøndelag County did not participate in the HUNT Study, and a further 14% of the participants did not have information on genotype or BMI and so were excluded from the analysis. In UK Biobank, only around 5% of people invited to take part were enrolled in the study. In addition to potential bias owing to differential selection, this means that the UK Biobank results are representative of healthier people than the UK population average. Genetic variants influence traits across the whole life course. Consequently, the associations we observe between genetically predicted BMI and mortality cannot be attributed to the causal effect of BMI at any particular period.^35^

Finally, our investigation was conducted in middle aged to early late aged participants of European descent based in Norway and the UK. Our findings might not be applicable to less healthy individuals, older people, and different nationalities and ethnicities.

### Conclusions and public health implications

Mendelian randomisation analyses in two population based prospective cohort studies suggested that population shifts to raise BMI across its distribution would lead to an overall increased risk of all cause mortality, but that increasing BMI to the normal weight category would reduce the risk of all cause mortality for underweight participants. The shape of the BMI-mortality curve was, however, different depending on sex, smoking status, age, and cause of death, with harm of having low BMI being evident in ever smokers, and an always-increasing relation between BMI and mortality in never smokers.

**What is already known on this topic**

Several large observational studies have shown a J shaped relation between body mass index (BMI) and all cause mortality
By using genetic variants in a mendelian randomisation approach, the shape of the BMI-mortality relation can be estimated in a way that is less susceptible to biases from reverse causation or confounding

**What this study adds**

Our mendelian randomisation analyses revealed a J shaped relation between genetically predicted BMI and the risk of all cause mortality risk, with the lowest risk at a BMI of around 22-25
Subgroup analyses stratified by smoking status suggested a J shaped relation in ever smokers, but an always-increasing relation of BMI with mortality in never smokers
In analyses split by cause of mortality (cardiovascular versus cancer versus non-cardiovascular non-cancer), a J shaped relation was only found for non-cardiovascular non-cancer mortality outcomes

## Data Availability

Data from the HUNT Study and UK Biobank are available on application.

## Web Extra

Extra material supplied by authors

Supplementary materials: Details of studies and participants, statistical methods, supplementary tables 1-9, and supplementary figures 1-4

## Notes

Contributors: YQS, SBu, and XMM contributed to the research questions and study design. XMM contributed to data collection for HUNT Study. YQS, SBu, and JRS conducted statistical analyses. YQS, SBu, and XMM interpreted the results and wrote the initial draft of the manuscript. JRS, SBe, AMW, SKK, QG, TRB, AMM, ASB, EDA, GÅV, JHB, JMK, and YC interpreted the data and helped to write the final draft of the manuscript. SB, YQS, and XMM had full access to the data in the study and can take responsibility for the integrity of the data and the accuracy of the data analysis. SBu and YQS are the guarantors. The corresponding author attests that all listed authors meet authorship criteria and that no others meeting the criteria have been omitted. YQS and SBu contributed equally to this work.

Transparency: The manuscripts guarantors (SBu and YQS) affirm that the manuscript is an honest, accurate, and transparent account of the study being reported; that no important aspects of the study have been omitted; and that any discrepancies from the study as planned have been explained.
