# Supplementary material for: Body mass index and all cause mortality in HUNT and UK Biobank studies: linear and non-linear mendelian randomisation analyses
Source: BMJ. 2019 Mar 26;364:l1042. doi: 10.1136/bmj.l1042 (PMC6434515; doi:10.1136/bmj.l1042)
Supplement: Supplementary file 1 — Supplementary materials: Details of studies and participants, statistical methods, supplementary tables 1-9, and supplementary figures 1-4 [file suny046273.ww1.pdf]

Supplementary Material

DETAILS OF STUDIES AND PARTICIPANTS

HUNT study

The Nord-Trøndelag Health Study (The HUNT Study) is a large population-based health survey conducted in Norway. The adult section of the HUNT study invited all inhabitants aged 20 years or older in the area of Nord-Trøndelag in three separate surveys: HUNT1 (1984–1986), HUNT2 (1995–1997) and HUNT3 (2006–2008). In the current study, we used data from HUNT2 in which 65,229 subjects participated (response rate 70%). All participants in HUNT2 were requested to complete a general questionnaire including health and lifestyle questions and social economic status. The HUNT Research Centre also received updated information about deaths of all causes and emigration of the HUNT participants from the Norwegian National Registry in which the dates of such events were recorded for all people living in Norway. The HUNT2 participants were followed up from their participation date until April 15, 2015 or the date of death.

We excluded subjects without data on body mass index (BMI) and BMI-related genetic variants, leaving 56,150 subjects in the analyses. Data of baseline variables were collected by questionnaires or clinical examination in HUNT2. These covariates were categorized as following: sex (men, women), pack-years of active smoking [0 (never smokers), former 0–10.0, former 10.1–20.0, former ≥20.1, current 0–10.0, current 10.1–20.0, current ≥20.1], alcohol consumption (never, 1–4, ≥5 times/month), physical activity (inactive, low, moderate, high), education (<10, 10–12, ≥13 years), economic difficulties (During the last year, has it at any time been difficult to meet the costs of food, transportation, housing and such? yes/no). The classification of each covariate has been widely used in the previous HUNT studies (1, 2). The HUNT study was approved by the Norwegian Regional Committees for Medical and Health Research Ethics. All participants gave their informed consent for participation in HUNT, and linkage to previous HUNT surveys and specific registries.

Measures of body mass index

At the clinical examination body weight and height were measured by trained nurses. Height and weight was measured with the participants wearing light clothes without shoes. Height was given in whole centimeters and weight was given in kilograms, rounded to the nearest half kilogram.

Measurement of genetic variants

DNA was isolated from blood samples collected in HUNT2 and was stored at the HUNT biobank. Genome-wide genotyping was carried out using Illumina HumanCoreExome arrays as described elsewhere (3). Imputation was performed on samples of recent European ancestry using Minimac3 (v2.0.1, http://genome.sph.umich.edu/wiki/Minimac3) (4) from a merged reference panel constructed from the Haplotype Reference Consortium panel (release version 1.1) (5) and a local reference panel based on 2201 whole-genome sequenced HUNT participants (6).

UK Biobank study

The UK Biobank (UKBB) cohort comprises just over 500,000 participants aged 40 to 69 years at baseline (94% of self-reported European ancestry) and recruited between 2006 and 2010 in 22 assessment centres throughout the UK. They were followed up for a variety of health conditions (7). An independent Ethics and Governance Council oversees adherence to the Ethics and Governance Framework and provides advice on the interests of research participants and the general public in relation to UKBB.

Individuals were excluded from analysis if height or weight measurements were absent, if they had non-European ancestry (self-report or judged by genetics), low call rate or excess heterozygosity (>3 standard deviations from the mean). Only one of each set of related individuals (third-degree relatives or closer) was included in analyses. Genetic variants were dropped from the analysis if they were not in Hardy—Weinberg equilibrium ($p < 10^{-5}$), had a call rate less than 99%, or an imputation score less than 0.9.

Measures of body mass index

Weight and height were collected at baseline when participants attended the initial assessment centre. Height (cm) was measured using a Seca 202 device in all participants in the UK Biobank. Weight (kg) was measured by a variety of means during the initial assessment centre visit, which was amalgamated into a single weight variable on the UK Biobank release data.

Measurement of genetic variants

Genome-wide genotyping was carried out using the Affymetrix UK BiLEVE Axiom array or the Affymetrix UK Biobank Axiom array for around 800,000 genetic variants. Imputation

was performed centrally by the Wellcome Trust Centre for Human Genetics using the Haplotype Reference Cohort.

Outcome definitions

Cardiovascular disease was defined as myocardial infarction, coronary artery disease, or any stroke. Cancer mortality was defined by International Classification of Disease [ICD] 10 code from C00 to D48. For analyses of non-fatal outcomes, anyone with a qualifying event before recruitment was excluded from analyses, and individuals were censored if they had a non-qualifying fatal event.

DETAILED DESCRIPTION OF STATISTICAL METHODS

Selection of genetic variants

In total, 77 genetic variants were reported as being associated with BMI at a genome-wide level of statistical significance ($p < 5\times10^{-8}$) in the Genetic Investigation of ANthropometric Traits (GIANT) consortium when restricting to participants of European ancestry (8). Two of these variants (rs12016871 and rs2033732) were not available in the HUNT study. We assessed the remaining 75 genetic variants for potential pleiotropy in the HUNT study by assessing associations with measured binary covariates: smoking (never/ever); alcohol consumption (times/month ≥1 vs. never); physical activity (active vs. inactive); education (years ≥10 vs. <10); and economic difficulties (yes vs. no) at a Bonferroni-corrected *p*-value threshold [$p < 6.7\times10^{-4}$ (0.05 divided by 75)]. Two SNPs (rs13021737 and rs16951275) were associated with smoking and therefore removed from subsequent analyses.

Generally speaking, assessment of pleiotropy is difficult due to two problems. First, multiple testing – when there are 75 variants and multiple potential pleiotropic pathways, an approach needs to balance the need to acknowledge multiple testing, but not to be overly conservative about detecting pleiotropy. Secondly, if a genetic variant is associated with a covariate due to a downstream effect of the exposure, this is not a violation of the instrumental variable assumptions (for example, if a genetic variant is associated with blood pressure solely due to its effect on BMI, then it is still a valid instrument). We chose a limited number of variables that we believed could be important confounders of the BMI—mortality relationship, and excluded genetic variants associated with one or more of these.

Allele score construction

The 73 variants included in the allele score are listed in Supplementary Table 1. All genetic variants are coded as 0, 1 or 2, corresponding to the number of copies of the effect allele (defined as the BMI-increasing allele). The weighted allele score for individual *i* is defined as:

$$\text{Weighted score}_i = \sum_{j=1,2,\ldots,J} \beta_j g_{ij}$$

where j indexes the genetic variants (from 1 to *J*), β_j are the weights, taken as the beta-coefficient associations from the GIANT consortium, and $g_{ij} = 0, 1, 2$ is the number of BMI-increasing alleles.

Linear Mendelian randomization

Linear Mendelian randomization for a single risk factor (univariable) is performed by regressing the exposure (BMI) on the allele score in a linear regression model to obtain the association estimate (beta-coefficient) β_X, and by regressing the outcome (all-cause mortality) on the allele score in a Cox proportional hazards regression model to obtain the association estimate (beta-coefficient) β_Y. The exposure regression model was adjusted for sex, age, age^2, and centre (in UK Biobank – there were 22 centres in total). The outcome regression model was adjusted for age and additionally for sex and centre in UK Biobank.

The Mendelian randomization estimate is obtained as the ratio $\frac{\beta_Y}{\beta_X}$, and its standard error as $\frac{se(\beta_Y)}{\beta_X}$ where $se(\beta_Y)$ is the standard error of the genetic association with the outcome.

We also calculated linear Mendelian randomization estimates within pre-defined categories of residual BMI: underweight (BMI < 18.5), normal weight 1 (BMI 18.5-19.9), normal weight 2 (BMI 20.0-24.9), overweight (BMI 25.0-29.9), and obese (BMI ≥ 30.0). Estimates were combined across the two studies by inverse-variance weighted meta-analysis within these pre-defined categories of BMI.

Non-linear Mendelian randomization

Statistical code for running non-linear Mendelian randomization is available at https://github.com/jrs95/nlmr. Here we use the *nlmr_summ* function to perform non-linear Mendelian randomization using summarized data. All non-linear analyses were conducted separately for HUNT and UK Biobank.

There are several reasons why we stratify the analysis on residual BMI rather than BMI as measured directly or another measure, such as the genetically predicted value of BMI. Residual BMI is defined as the residual from the regression of BMI on the allele score, and represents the predicted BMI value for an individual if their allele score took the value zero. First, if we stratified on BMI, we would induce a bias due to overadjustment. This is because BMI is on the causal pathway from the genetic variants to the outcome (9). Secondly, we could stratify on the genetically predicted value of BMI, which is numerically equivalent to the allele score for BMI. However, as such a small proportion of variance in BMI is explained by the genetic variants, individuals in UK Biobank in the lowest decile of the gene score have an average BMI of around 26.5, whereas individuals in the highest decile of the gene score have an average BMI of around 28.5. Stratifying the population based on the gene score would not allow us to investigate a wide enough range of the BMI distribution to give a meaningful result.

By stratifying on residual BMI, we avoid overadjustment bias and collider bias, as residual BMI is not downstream of the genetic variants in a causal diagram (9). We calculate causal estimates in strata of individuals who would have BMI values in the same category if they had the same genotype. As the scale of residual BMI is similar to that of untransformed BMI, we are able to consider estimates for strata that span a wide range of BMI values, but without inducing the bias that would occur if we stratified on BMI directly.

To calculate residual BMI, we first regress BMI on the allele score to obtain fitted values of genetically-predicted BMI. We then subtract genetically-predicted BMI from BMI to obtain residual BMI. The allele score is centred to have mean 0, so that subtracting residual BMI does not influence the average BMI in the studies.

Next, we divide the population in 100 equal groups based on residual BMI, and then calculate genetic associations with the outcome in each group. The genetic association with the exposure is assumed to be constant for all individuals, and a single coefficient is estimated using the totality of the dataset. We assessed this assumption by estimating the genetic association with BMI in each stratum of the population and applying the non-linearity tests detailed below – there was no evidence for the departure from this assumption for the genetic associations with the exposure aside from estimates in the top and bottom quantiles.

We divide the outcome regression coefficient by the exposure regression coefficient as in a linear Mendelian randomization analysis to obtain local average causal effects (LACE) in

each stratum. The same adjustment is performed as in the linear Mendelian randomization analyses. We then performed meta-regression of the LACE estimates against the mean of the exposure in each stratum in a flexible semiparametric framework using the derivative of fractional polynomial models of degrees 1 and 2. Two tests for non-linearity were reported: a fractional polynomial test, which assesses whether a non-linear model fits the data better than a linear model; and a trend test, which performs meta-regression of the LACE estimates on the mean BMI in each stratum. This same trend test is performed on the categorized Mendelian randomization estimates in Table 2 of the main manuscript.

As a technical note, all comparisons are conducted within strata, so that the graph provides information on the expected average change in the outcome if a person with a BMI value of (say) 25 kg/m^2 instead had a BMI value of 26 kg/m^2. Inferences on the expected change in the outcome if that person instead had a BMI value of (say) 40 kg/m^2 can only be made by assuming that those in the population with a BMI of 25 kg/m^2 would be similar to those in the population with a BMI value of 40 kg/m^2 if their BMI were increased.

Before fitting the model, we subtracted 18 from values of BMI, as the fractional polynomials require positive values of the risk factor close to zero to obtain the most flexible model fit. The reference value for BMI was taken as 25 kg/m^2. We fit all possible fractional polynomials of degree 1 and 2, using powers -2, -1, 0 (logarithm), 0.5, 1, 2, and 3, as described in (10), to find the best-fitting polynomials of degree 1 and 2. The fractional polynomial of degree 2 is fit to the data, unless the fractional polynomial of degree 1 as good of a fit ($p > 0.5$), as judged by the likelihood ratio test. The rationale for preferring fractional polynomials of degree 2 was to allow a more detailed shape in the BMI—mortality relationship.

Collider bias

Collider bias occurs when considering the association between two variables while conditioning on a third variable. If the two variables are both causes of the third (causal arrows go from the first two variables into the third, hence “collider”), then conditioning on the third variable leads to a conditional association between the two variables, even if they were not associated marginally. In the assumptions of Mendelian randomization, the genetic variants are independently distributed from confounders. Even if the two variables are independent marginally (i.e. in the population as a whole), they may become correlated if we

condition on a collider, leading to bias in the analysis (collider bias, also known as selection bias).

In general, conditioning on a variable in Mendelian randomization leads to collider bias if it is a function of the outcome, or if it is a function of the risk factor. Conditioning does not lead to bias if it is a function of confounders only, nor if it is a function of the outcome only under the causal null (11). However our investigation, smoking is a function of the risk factor, as individuals with higher BMI are more likely to smoke. Carreras-Torres et al (12) showed that a standard deviation increase in BMI led to a 18% increase in the risk of being a smoker in the UK Biobank (OR 1.18, 95% CI 1.13 to 1.23).

We have investigated collider bias in Mendelian randomization in great detail in a recent publication (13), and shown using a simulation study that bias attributable to an effect of the risk factor on the stratifying variable of this magnitude (an odds ratio of 1.18 per standard deviation increase in the exposure) leads to negligible bias in Mendelian randomization estimates. In this case, the distortion in estimates due to stratifying on a collider is not enough to lead to such marked differences in the Mendelian randomization estimates calculated in ever-smokers and never-smokers.

Supplementary Table 1. Genetic variants and associations with BMI based on European sex-combined analysis from GIANT (8). Beta-coefficents are in standard deviation units.

SNP	Effect allele*	Other allele	Beta-coefficient	Standard error of beta	Number of subjects	*P*-value
rs1558902	A	T	0.082	0.003	320 073	7.51E-153
rs6567160	C	T	0.056	0.004	321 958	3.93E-53
rs10938397	G	A	0.040	0.003	320 955	3.21E-38
rs543874	G	A	0.048	0.004	322 008	2.62E-35
rs2207139	G	A	0.045	0.004	322 019	4.13E-29
rs11030104	A	G	0.041	0.004	322 103	5.56E-28
rs3101336	C	T	0.033	0.003	316 872	2.66E-26
rs7138803	A	G	0.032	0.003	322 092	8.15E-24
rs10182181	G	A	0.031	0.003	321 759	8.78E-24
rs3888190	A	C	0.031	0.003	321 930	3.14E-23
rs1516725	C	T	0.045	0.005	320 644	1.89E-22
rs12446632	G	A	0.040	0.005	316 758	1.48E-18
rs2287019	C	T	0.036	0.004	300 921	4.59E-18
rs3817334	T	C	0.026	0.003	321 959	5.15E-17
rs2112347	T	G	0.026	0.003	322 019	6.19E-17
rs12566985	G	A	0.024	0.003	319 282	3.28E-15
rs3810291	A	G	0.028	0.004	296 261	4.81E-15
rs7141420	T	C	0.024	0.003	321 970	1.23E-14
rs13078960	G	T	0.030	0.004	322 135	1.74E-14
rs10968576	G	A	0.025	0.003	322 061	6.61E-14
rs17024393	C	T	0.066	0.009	297 874	7.03E-14
rs657452	A	G	0.023	0.003	313 651	5.48E-13
rs12429545	A	G	0.033	0.005	312 934	1.09E-12
rs12286929	G	A	0.022	0.003	321 903	1.31E-12
rs13107325	T	C	0.048	0.007	321 461	1.83E-12
rs11165643	T	C	0.022	0.003	320 730	2.07E-12
rs7903146	C	T	0.023	0.003	322 130	1.11E-11
rs10132280	C	A	0.023	0.003	321 797	1.14E-11
rs17405819	T	C	0.022	0.003	322 085	2.07E-11
rs1016287	T	C	0.023	0.003	321 969	2.25E-11
rs4256980	G	C	0.021	0.003	320 028	2.90E-11
rs17094222	C	T	0.025	0.004	321 770	5.94E-11
rs12401738	A	G	0.021	0.003	322 070	1.15E-10
rs7599312	G	A	0.022	0.003	322 024	1.17E-10
rs2365389	C	T	0.020	0.003	316 768	1.63E-10
rs205262	G	A	0.022	0.004	315 542	1.75E-10
rs2820292	C	A	0.020	0.003	321 707	1.83E-10
rs12885454	C	A	0.021	0.003	320 823	1.94E-10
rs16851483	T	G	0.048	0.008	233 929	3.55E-10
rs1167827	G	A	0.020	0.003	306 238	6.33E-10
rs758747	T	C	0.023	0.004	308 688	7.47E-10
rs1928295	T	C	0.019	0.003	321 979	7.91E-10
rs9925964	A	G	0.019	0.003	318 385	8.11E-10

rs11126666	A	G	0.021	0.003	321 979	1.33E-09
rs2650492	A	G	0.021	0.004	319 464	1.92E-09
rs6804842	G	A	0.019	0.003	321 463	2.48E-09
rs12940622	G	A	0.018	0.003	322 032	2.49E-09
rs11847697	T	C	0.049	0.008	306 243	3.99E-09
rs4740619	T	C	0.018	0.003	321 887	4.56E-09
rs13191362	A	G	0.028	0.005	321 902	7.34E-09
rs3736485	A	G	0.018	0.003	321 398	7.41E-09
rs17001654	G	C	0.031	0.005	233 722	7.76E-09
rs11191560	C	T	0.031	0.005	321 893	8.45E-09
rs1528435	T	C	0.018	0.003	321 924	1.20E-08
rs2075650	A	G	0.026	0.005	308 408	1.25E-08
rs1000940	G	A	0.019	0.003	321 836	1.28E-08
rs2033529	G	A	0.019	0.003	321 917	1.39E-08
rs11583200	C	T	0.018	0.003	322 095	1.48E-08
rs9400239	C	T	0.019	0.003	321 988	1.61E-08
rs10733682	A	G	0.017	0.003	320 727	1.83E-08
rs11688816	G	A	0.017	0.003	322 051	1.89E-08
rs11057405	G	A	0.031	0.006	314 111	2.02E-08
rs2121279	T	C	0.025	0.004	322 065	2.31E-08
rs29941	G	A	0.018	0.003	321 970	2.41E-08
rs11727676	T	C	0.036	0.006	296 401	2.55E-08
rs3849570	A	C	0.019	0.003	284 339	2.60E-08
rs6477694	C	T	0.017	0.003	322 048	2.67E-08
rs7899106	G	A	0.040	0.007	321 770	2.96E-08
rs2176598	T	C	0.020	0.004	316 848	2.97E-08
rs2245368	C	T	0.032	0.006	205 675	3.19E-08
rs17724992	A	G	0.019	0.004	319 588	3.42E-08
rs7243357	T	G	0.022	0.004	322 107	3.86E-08
rs1808579	C	T	0.017	0.003	322 032	4.17E-08

*BMI-increasing allele

Supplementary Table 2. Distribution of body mass index in HUNT and UK Biobank studies

Body mass index (kg/m^2)	HUNT (n=56,150)	UK Biobank (n=366,385)
Percentile 2.5%	19.8	20.1
Percentile 5%	20.7	20.9
Percentile 25%	23.6	24.1
Percentile 50%	25.8	26.7
Percentile 75%	28.6	29.8
Percentile 95%	33.7	36.1
Percentile 97.5%	35.9	38.8

Supplementary Table 3: Linear Mendelian randomization estimates from robust methods (MR-Egger and weighted median)

	HUNT	UK Biobank
Primary method (allele score)	1.03 (1.00 to 1.06)<br>p = 0.093	1.05 (1.02 to 1.09)<br>p = 0.002
MR-Egger method	0.99 (0.93 to 1.04)<br>p = 0.61	1.08 (1.03 to 1.13)<br>p = 0.003
Weighted median method	0.99 (0.95 to 1.04)<br>p = 0.78	1.07 (1.00 to 1.16)<br>p = 0.064

Supplementary Table 4: Linear Mendelian randomization estimates – hazard ratio (95% confidence interval) for all-cause mortality per 1 kg/m^2 increase in body mass index in never-smokers and ever-smokers

In HUNT	Never-smokers (n = 23,686)	Ever-smokers (n = 31,395)
Overall	1.07 (1.01 to 1.13), p = 0.02	0.99 (0.95 to 1.03), p = 0.71
Within residual BMI categories:		
- Underweight (<18.5 kg/m^2)	1.23 (0.58 to 2.60), p = 0.59	0.59 (0.38 to 0.92), p = 0.020
- Normal weight 1 (18.5-19.9 kg/m^2)	1.34 (0.79 to 2.28), p = 0.28	0.78 (0.59 to 1.02), p = 0.068
- Normal weight 2 (20.0-24.9 kg/m^2)	1.07 (0.96 to 1.19), p = 0.26	0.94 (0.87 to 1.01), p = 0.077
- Overweight (25.0-29.9 kg/m^2)	1.05 (0.97 to 1.14), p = 0.19	1.03 (0.97 to 1.09), p = 0.35
- Obese (≥30.0 kg/m^2)	1.08 (0.96 to 1.21), p = 0.18	1.00 (0.92 to 1.10), p = 0.92
Trend test p-value	0.93	0.06
**In UK Biobank**	**Never-smokers (n = 197,310)**	**Ever-smokers (n = 169,075)**
Overall	1.05 (0.99 to 1.11), p = 0.11	1.04 (1.00 to 1.08), p = 0.031
Within residual BMI categories:		
- Underweight (<18.5 kg/m^2)	0.69 (0.37 to 1.28), p = 0.24	0.59 (0.41 to 0.85), p = 0.004
- Normal weight 1 (18.5-19.9 kg/m^2)	1.16 (0.78 to 1.73), p = 0.46	0.64 (0.49 to 0.83), p = 0.001
- Normal weight 2 (20.0-24.9 kg/m^2)	1.00 (0.90 to 1.11), p = 0.97	1.00 (0.93 to 1.08), p = 0.93
- Overweight (25.0-29.9 kg/m^2)	1.05 (0.96 to 1.15), p = 0.25	1.03 (0.97 to 1.09), p = 0.33
- Obese (≥30.0 kg/m^2)	1.08 (0.97 to 1.19), p = 0.17	1.11 (1.04 to 1.19), p = 0.002
Trend test p-value	0.31	0.012

Low p-values for the trend test suggest a non-linear trend in the association between genetically-predicted BMI and all-cause mortality. The test uses meta-regression to assess whether the local average causal effect (LACE) estimates vary as a function of the mean BMI in each category.

Supplementary Table 5. Information on all-cause mortality in subgroups in the HUNT study (n=56150)

Subgroups	Subjects	Deaths (all-cause)	Percentage of death
Sex			
Men	26,447	6318	23.9
Women	29,703	5697	19.2
Smoking groups			
Never smokers	23,686	4314	18.2
Ever smokers	31,395	7226	23.0
Age at risk*			
<65 years	44,176	1277	2.9
≥65 years	30,003	10738	35.8

*Some subjects were included in both groups

1069 individuals had unknown smoking status and so were omitted from the smoking-stratified analyses.

Supplementary Table 6. Disease outcomes in UK Biobank (including men/women, never/ever smokers, younger/older)

Subgroups	Subjects	Deaths (all-cause)	Cardiovascular deaths (%)	Cancer deaths (%)	Other deaths (%)
Sex					
Men	168,157	6290	1598 (25)	3351 (53)	1290 (21)
Women	198,228	4054	547 (13)	2774 (68)	708 (17)
Smoking groups					
Never smokers	197,310	3800	703 (19)	2344 (62)	725 (19)
Ever smokers	169,075	6544	1442 (22)	3781 (58)	1273 (19)
Age at risk*					
<65 years	296,147	3752	725 (19)	2216 (59)	758 (20)
≥65 years	189,413	6590	1419 (22)	3908 (59)	1239 (19)

*Some subjects were included in both groups

Numbers do not add up perfectly across rows and columns as there were 2 individuals with a missing age at death, and 76 with a missing cause of death.

Supplementary Table 7. *P*-values from non-linearity tests

	Trend test		Fractional polynomial non-linearity	
	HUNT	UK Biobank	HUNT	UK Biobank
Overall	0.11	0.0008	0.06	0.009
Men	0.49	0.037	0.01	0.10
Women	0.23	0.012	0.26	0.044
Never-smokers	0.47	0.51	0.54	0.51
Ever-smokers	0.02	<0.001	<0.001	0.004
Age at risk < 65 years	0.79	0.012	0.66	0.034
Age at risk ≥ 65 years	0.16	0.021	0.15	0.033

Low p-values for the trend test suggest a non-linear trend in the association between genetically-predicted BMI and all-cause mortality. The test uses meta-regression to assess whether the local average causal effect (LACE) estimates vary as a function of the mean BMI in each stratum. Low p-values in the fractional polynomial non-linearity test suggest a non-linear model fits better than a linear model.

Supplementary Table 8. Hazard ratios in percentiles of the population stratified by residual BMI in HUNT. Reference category is 25 kg/m^2. For each percentile, we provide the BMI value, hazard ratio (HR) and 95% confidence interval (CI).

	Overall			Never-smokers			Ever-smokers		
Percentile	BMI	HR	95% CI	BMI	HR	95% CI	BMI	HR	95% CI
1st	18.8	1.17	(0.90 to 1.53)	19.0	0.77	(0.65 to 0.92)	18.6	1.88	(1.34 to 2.64)
5th	20.7	1.01	(0.87 to 1.17)	20.8	0.80	(0.68 to 0.93)	20.6	1.28	(1.06 to 1.55)
10th	21.7	0.99	(0.88 to 1.10)	21.8	0.82	(0.72 to 0.94)	21.6	1.17	(1.01 to 1.34)
20th	23.0	0.98	(0.92 to 1.04)	23.1	0.88	(0.80 to 0.96)	23.0	1.07	(0.98 to 1.16)
30th	24.0	0.99	(0.95 to 1.02)	24.0	0.93	(0.88 to 0.98)	24.0	1.03	(0.99 to 1.07)
40th	25.0	1.00	(1.00 to 1.00)	25.0	1.00	(1.00 to 1.00)	24.9	1.00	(1.00 to 1.01)
50th	25.8	1.02	(0.99 to 1.04)	25.9	1.08	(1.03 to 1.14)	25.8	0.99	(0.95 to 1.02)
60th	26.8	1.04	(0.98 to 1.11)	26.9	1.19	(1.06 to 1.34)	26.8	0.98	(0.90 to 1.05)
70th	27.9	1.08	(0.98 to 1.19)	28.0	1.33	(1.10 to 1.61)	27.8	0.97	(0.86 to 1.10)
80th	29.3	1.13	(0.98 to 1.31)	29.4	1.54	(1.15 to 2.08)	29.2	0.98	(0.81 to 1.18)
90th	31.6	1.25	(0.99 to 1.58)	31.7	1.95	(1.21 to 3.14)	31.4	1.01	(0.75 to 1.34)
95th	33.7	1.37	(1.00 to 1.89)	33.9	2.32	(1.20 to 4.49)	33.5	1.05	(0.71 to 1.55)
99th	38.8	1.80	(1.05 to 3.06)	39.1	2.17	(0.59 to 8.00)	38.5	1.23	(0.64 to 2.35)

Supplementary Table 9. Hazard ratios in percentiles of the population stratified by residual BMI in UK Biobank. Reference category is 25 kg/m^2. For each percentile, we provide the BMI value, hazard ratio (HR) and 95% confidence interval (CI).

	Overall			Never-smokers			Ever-smokers		
Percentile	BMI	HR	95% CI	BMI	HR	95% CI	BMI	HR	95% CI
1st	19.1	1.39	(1.02 to 1.90)	19.0	0.93	(0.81 to 1.06)	19.2	1.71	(1.18 to 2.46)
5th	20.9	1.11	(0.94 to 1.32)	20.8	0.94	(0.83 to 1.06)	21.2	1.23	(1.01 to 1.49)
10th	22.1	1.04	(0.93 to 1.17)	21.9	0.94	(0.85 to 1.05)	22.3	1.10	(0.98 to 1.25)
20th	23.5	1.00	(0.95 to 1.05)	23.3	0.96	(0.90 to 1.03)	23.8	1.02	(0.97 to 1.08)
30th	24.6	1.00	(0.98 to 1.01)	24.3	0.98	(0.96 to 1.01)	25.0	1.00	(1.00 to 1.00)
40th	25.7	1.01	(0.99 to 1.03)	25.4	1.01	(0.99 to 1.03)	26.0	1.00	(0.96 to 1.04)
50th	26.7	1.04	(0.99 to 1.10)	26.4	1.04	(0.97 to 1.11)	27.1	1.02	(0.94 to 1.11)
60th	27.8	1.09	(1.00 to 1.19)	27.4	1.08	(0.95 to 1.23)	28.2	1.06	(0.94 to 1.20)
70th	29.1	1.17	(1.02 to 1.33)	28.7	1.14	(0.92 to 1.41)	29.4	1.14	(0.96 to 1.35)
80th	30.7	1.31	(1.08 to 1.58)	30.4	1.24	(0.89 to 1.72)	31.1	1.27	(1.00 to 1.61)
90th	33.4	1.65	(1.23 to 2.21)	33.2	1.45	(0.84 to 2.49)	33.7	1.62	(1.12 to 2.32)
95th	36.1	2.17	(1.44 to 3.28)	35.9	1.73	(0.81 to 3.71)	36.4	2.17	(1.32 to 3.59)
99th	42.3	4.81	(2.29 to 10.11)	42.2	2.68	(0.66 to 10.92)	42.6	5.26	(2.16 to 12.79)

Supplementary Figure 1: Scatter plot of genetic associations with the risk factor (horizontal axis, kg/m^2) and with the outcome (vertical axis, odds ratios). Outlying points on the graph may represent pleiotropic genetic variants.

A) HUNT:

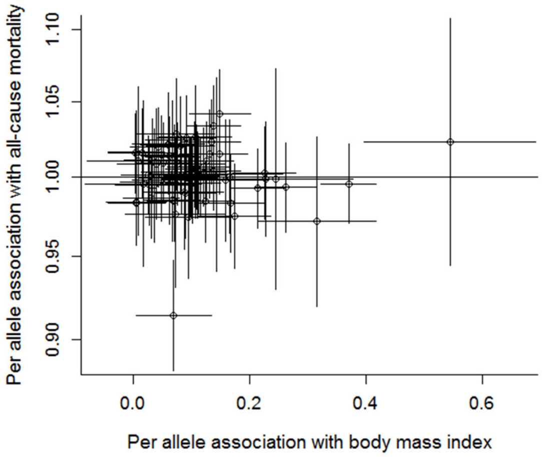

B) UK Biobank:

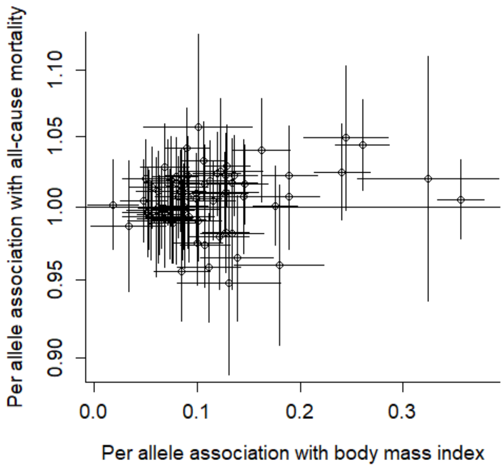

Supplementary Figure 2: Non-linear Mendelian randomization excluding individuals who died in the first 2 years of follow-up – dose-response curve between body mass index and all-cause mortality. A) HUNT data (738 individuals excluded); B) UK Biobank (1919 individuals excluded). Gradient at each point of the curve is the localized average causal effect. Grey lines represent 95% confidence intervals.

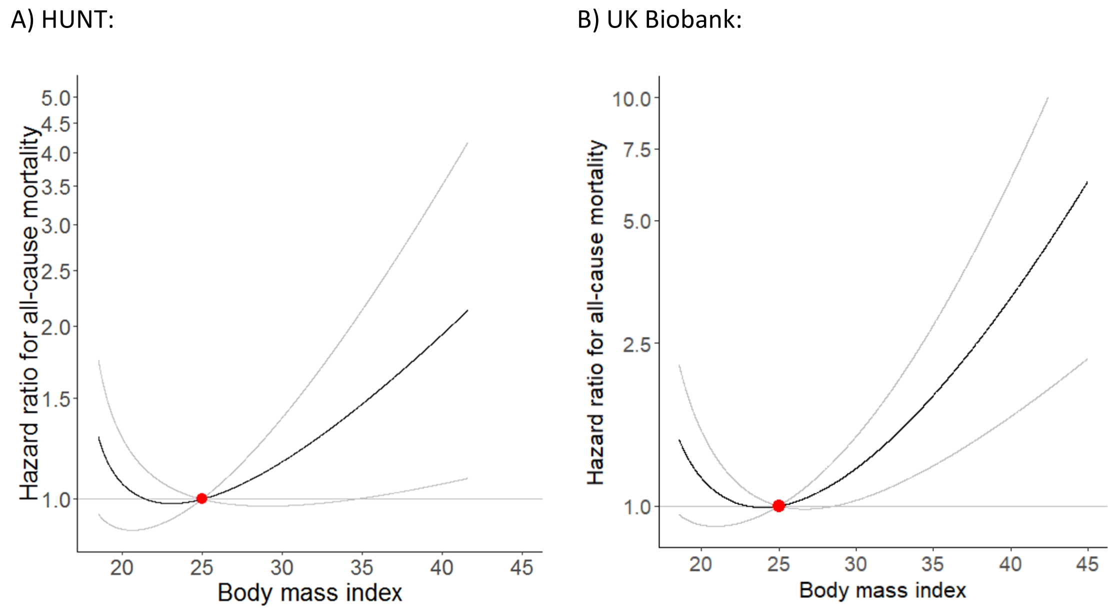

Supplementary Figure 3: Non-linear Mendelian randomization – dose-response curve between body mass index and all-cause mortality in young and old subjects. A) HUNT, age at risk up to 65; B) HUNT, age at risk 65 and over; C) UK Biobank, age at risk up to 65; D) UK Biobank, age at risk 65 and over. Gradient at each point of the curve is the localized average causal effect. Grey lines represent 95% confidence intervals.

A) HUNT, age at risk up to 65:

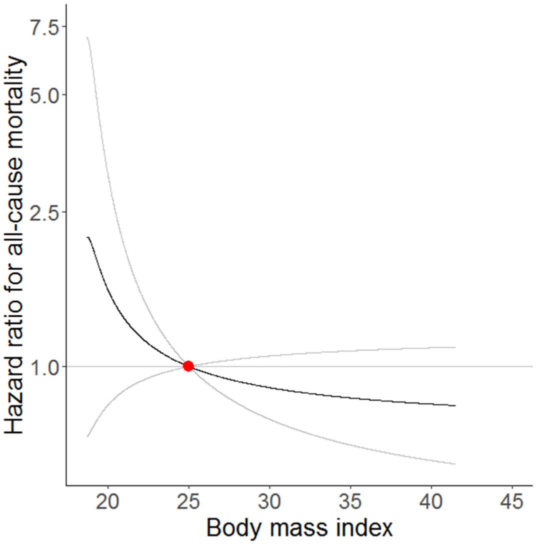

B) HUNT, age at risk 65 and over:

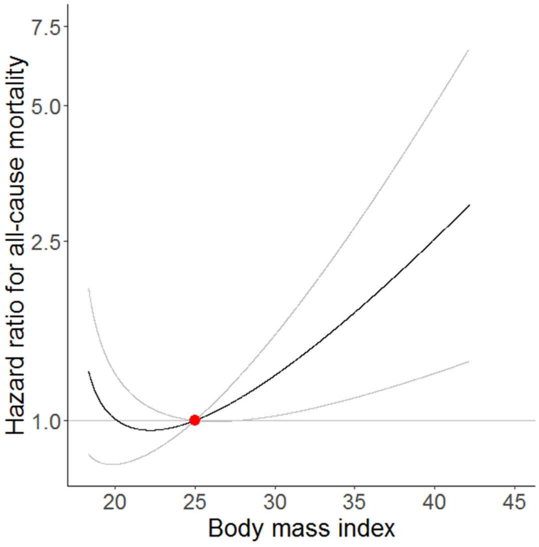

C) UK Biobank, age at risk up to 65:

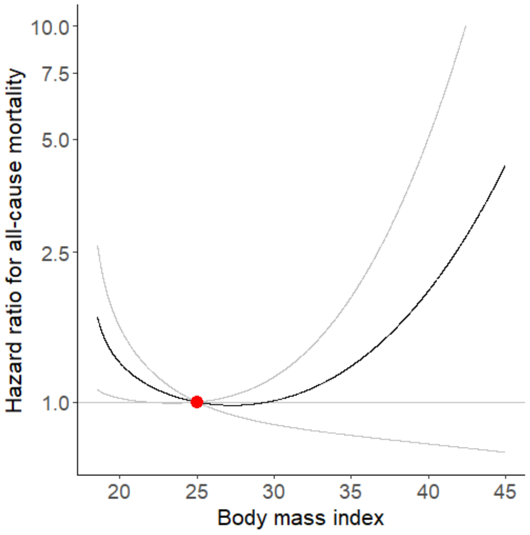

D) UK Biobank, age at risk 65 and over:

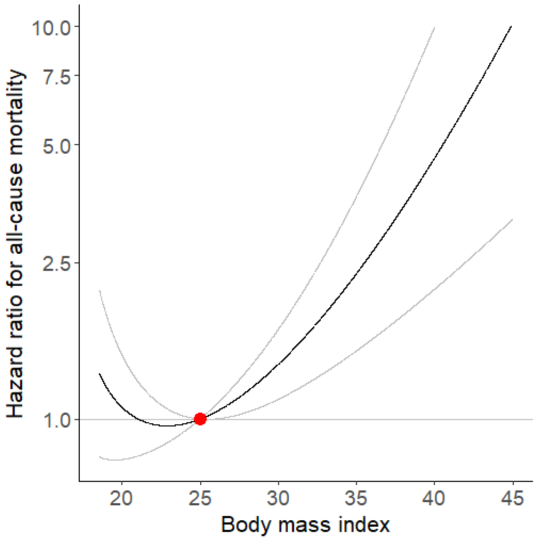

Supplementary Figure 4: Non-linear Mendelian randomization in UK Biobank – dose-response curve between body mass index and incident disease. A) Any cardiovascular disease; B) Any cancer. Gradient at each point of the curve is the localized average causal effect.

A: Any cardiovascular disease B: Any cancer

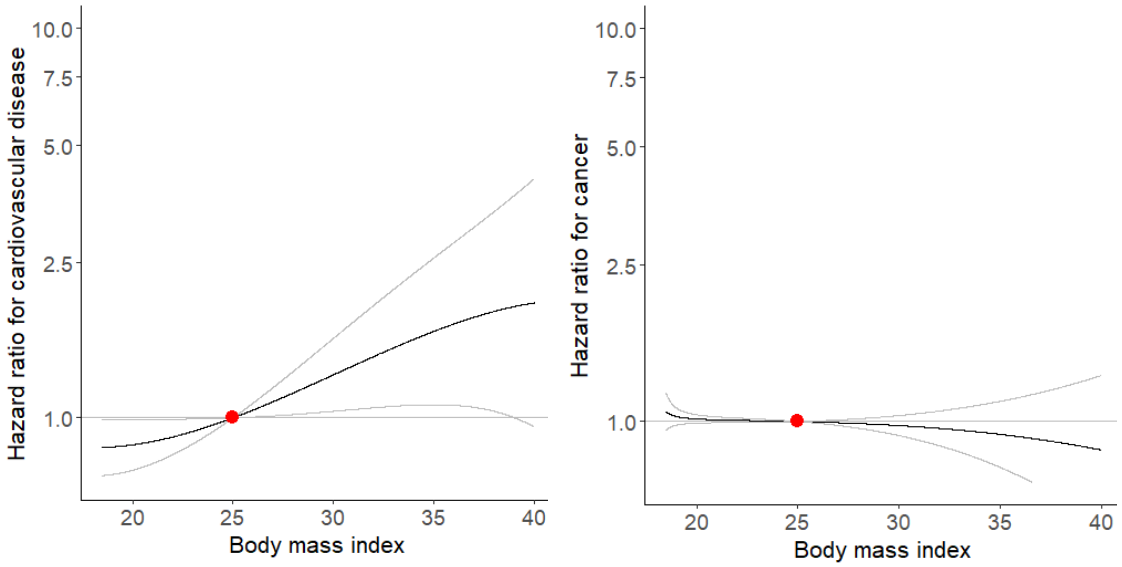

References

1. Sun YQ, Chen Y, Langhammer A, Skorpen F, Wu C, Mai XM. Passive smoking in relation to lung cancer incidence and histologic types in Norwegian adults: the HUNT study. Eur Respir J. 2017;50(4). doi:10.1183/13993003.00824-2017

2. Sun YQ, Langhammer A, Skorpen F, Chen Y, Mai XM. Serum 25-hydroxyvitamin D level, chronic diseases and all-cause mortality in a population-based prospective cohort: the HUNT Study, Norway. BMJ Open. 2017;7(6):e017256. doi:10.1136/bmjopen-2017-017256

3. Ferreira MA, Vonk JM, Baurecht H, et al. Shared genetic origin of asthma, hay fever and eczema elucidates allergic disease biology. Nat Genet. 2017. doi:10.1038/ng.3985

4. Das S, Forer L, Schonherr S, et al. Next-generation genotype imputation service and methods. Nat Genet. 2016;48(10):1284-7. doi:10.1038/ng.3656

5. McCarthy S, Das S, Kretzschmar W, et al. A reference panel of 64,976 haplotypes for genotype imputation. Nat Genet. 2016;48(10):1279-83. doi:10.1038/ng.3643

6. Zhou W, Fritsche LG, Das S, et al. Improving power of association tests using multiple sets of imputed genotypes from distributed reference panels. Genet Epidemiol. 2017;41(8):744-55. doi:10.1002/gepi.22067

7. Sudlow C, Gallacher J, Allen N, et al. UK biobank: an open access resource for identifying the causes of a wide range of complex diseases of middle and old age. PLoS Med. 2015;12(3):e1001779. doi:10.1371/journal.pmed.1001779

8. Locke AE, Kahali B, Berndt SI, et al. Genetic studies of body mass index yield new insights for obesity biology. Nature. 2015;518(7538):197-206. doi:10.1038/nature14177

9. Burgess S, Davies NM, Thompson SG. Instrumental variable analysis with a nonlinear exposure-outcome relationship. Epidemiology. 2014;25(6):877-85. doi:10.1097/ede.0000000000000161

10. Staley JR, Burgess S. Semiparametric methods for estimation of a nonlinear exposure-outcome relationship using instrumental variables with application to Mendelian randomization. Genet Epidemiol. 2017;41(4):341-52. doi:10.1002/gepi.22041

11. Hughes RA, Davies NM, Davey Smith G, Tilling K. Selection bias in instrumental variable analyses. bioRxiv. 2017. doi:10.1101/192237

12. Carreras-Torres R, Johansson M, Haycock PC, et al. Role of obesity in smoking behaviour: Mendelian randomisation study in UK Biobank. BMJ. 2018;361. doi:10.1136/bmj.k1767

13. Gkatzionis A, Burgess S. Contextualizing selection bias in Mendelian randomization: how bad is it likely to be? International Journal of Epidemiology. 2018.
